# Pulsed-Laser-Deposited LiMn_2_O_4_ Thin-Film Solid-State Microbatteries with Extended Voltage Window Cycling

**DOI:** 10.1021/acsaem.5c03983

**Published:** 2026-03-17

**Authors:** Juan Carlos Gonzalez-Rosillo, Jędrzej Morzy, Yaroslav E. Romanyuk, Moritz H. Futscher, Albert Tarancón, Alex Morata

**Affiliations:** † Department of Advanced Materials for Energy Applications, Catalonia Institute for Energy Research (IREC), Jardins de les Dones de Negre 1, 08930 Sant Adrià del Besòs (Barcelona), Spain; ‡ Laboratory for Thin Films and Photovoltaics, Empa − Swiss Federal Laboratories for Materials Science and Technology, Überlandstrasse 129, 8600 Dübendorf, Switzerland; § Catalan Institution for Research and Advanced Studies (ICREA), Passeig Lluís Companys 23, 08010 Barcelona, Spain

**Keywords:** solid-state batteries, thin film batteries, pulsed laser deposition, extended voltage window cycling, cathode-electrolyte interface, microbatteries

## Abstract

Thin-film microbatteries provide on-chip and surface-mount energy storage for Si-based microsystems, where device area is the primary constraint. Commercial implementations, available for more than 20 years, have largely relied on LiCoO_2_ cathodes because they are straightforward to process and package. LiMn_2_O_4_ offers a cobalt-free alternative; however, in conventional liquid-electrolyte Li-ion cells, its use is constrained by Mn dissolution and capacity fade, especially when the voltage window is widened to access its theoretical capacity of ∼119 μAh·cm^–2^·μm^–1^ (∼296 mAh·g^–1^). Thin-film solid-state architectures can mitigate these limitations and are naturally aligned with footprint-limited applications, where areal capacity and areal energy are the relevant figures of merit. The focus of this study is to examine the device behavior of LiMn_2_O_4_ thin-film microbatteries operated in a wider voltage window, using a LiPON solid electrolyte and a Li metal anode. Polycrystalline LiMn_2_O_4_ cathodes (∼850 nm) were grown by pulsed laser deposition with sequential Li_2_O enrichment during growth. X-ray diffraction, Raman features, and depth-profiling glow discharge optical emission spectroscopy are consistent with the presence of a Li-rich spinel component formed during deposition. The resulting LiMn_2_O_4_/LiPON/Li cells, cycled between 2.0 and 4.5 V, deliver up to ∼50 μAh·cm^–2^ at low rates; at higher rates, the wider window enables capacities up to ∼4 times those obtained on the same devices in the conventional 3.5–4.5 V window. Impedance measurements are used to track evolution during conditioning and operation. Finally, we provide an overview of relevant LiMn_2_O_4_ solid-state thin-film microbatteries and outline a tentative route to stabilize the LiMn_2_O_4_/LiPON interface under wider-window operation.

## Graphical abstract

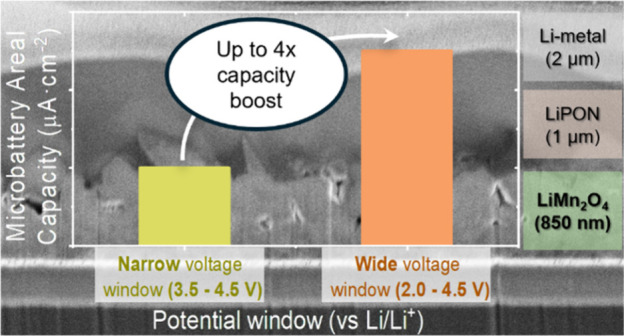

## Introduction

Miniaturized IoT, wearable, and implantable systems require energy storage that fits a fixed footprint, integrates on-chip with microelectronics, and remains compatible with standard workflows within the semiconductor industry.^1^ Thin-film microbatteries meet these requirements using solid electrolytes and micrometre-scale cathodes.^2,3^ They can be fabricated directly on Si chips or supplied as components for conventional assembly. The technology has been commercially available for more than two decades, predominantly with LiPON electrolytes and LiCoO_2_ cathodes. The device performance in this area-limited context is most meaningfully expressed as areal capacity (μAh·cm^–2^), areal energy (μWh·cm^–2^), and areal power density (mW·cm^–2^). With appropriate orientation control, LiCoO_2_ films have even been demonstrated to reach ≈1.2 mAh·cm^–2^ at ∼30 μm thickness in solid-state cells.^4^

Within this context, LiMn_2_O_4_ poses an attractive, cobalt-free alternative to LiCoO_2_. First reported in the 1980s, LiMn_2_O_4_ combines a relatively high operating voltage (4.2 V) with a spinel framework that supports three-dimensional Li diffusion with the use of abundant manganese.^5^ In the conventional voltage range (3.5–4.5 V vs Li/Li^+^), LiMn_2_O_4_ has a theoretical capacity of ∼59 μAh·cm^–2^·μm^–1^ (148 mAh·g^–1^), though practical capacities of approximately ∼48 μAh·cm^–2^·μm^–1^ (120 mAh·g^–1^) are typically achieved.^5^ Extending the cycling range to 2.0–4.5 V allows access to an additional plateau near 3 V, corresponding to the formation of the Li_2_Mn_2_O_4_ phase. This extended range effectively doubles the theoretical capacity to ∼119 μAh·cm^–2^·μm^–1^ (296 mAh·g^–1^), enabling significantly higher energy density than, for instance, LiCoO_2_ (∼138 mAh·g^–1^, 69 μAh·cm^–2^·μm^–1^). However, this comes at the cost of severe manganese dissolution (in liquid electrolytes) due to the formation of Mn^2+^,^5–8^ as well as substantial volume changes, and particle cracking associated with the lithiation process.^9^ Only in very thin films (<200 nm, low areal capacity), several studies with liquid electrolytes have shown that a wider voltage window can be accessed for few tens of cycles approaching the theoretical capacity of the Li_2_Mn_2_O_4_ phase.^10–12^ In particular, Zheng et al. reported that strain/orientation control can stabilize Li_1+x_Mn_2_O_4_ and improve utilization; however, such approaches typically rely on epitaxial or single-crystal growth and are difficult to translate to scalable polycrystalline processes.^13^

A representative survey of solid-state LiMn_2_O_4_ thin film batteries spanning over 25 years is summarized in Table S1 (Supporting Information, Section I), including fabrication methods, cycling conditions and voltage range, film thicknesses, and achieved areal capacities. Early studies using e-beam evaporation and RF sputtering followed by high-temperature annealing established crystalline LiMn_2_O_4_ operation but frequently reported limited areal capacity in the conventional narrow voltage window, especially for films >1 μm where diffusion constraints dominate.^14–17^ Amorphous counterparts generally showed lower energy storage capabilities,^14,18,19^ while 3D architectures^20,21^ and composite cathodes^22^ have shown promising results in improved cycling stability and rate capability. Despite these advancements, the near-full capacity utilization reported in early studies in the field^23^ has proved difficult to be reproduced broadly, and even recent reports indicate that LiMn_2_O_4_/LiPON interface can evolve under cycling even within the narrower voltage window.^24^ Because thickness, porosity, current density, and voltage range differ across studies, device-to-device comparisons should be made cautiously. However, a consistent trend emerges from our survey: increasing the thickness above 500 nm of planar, polycrystalline LiMn_2_O_4_ while maintaining high cathode utilization is challenging, whereas exploration of wider-window operation in solid-state cells remains comparatively understudied and may offer a practical lever to improve areal capacity at the device level.

In this work, we examine Pulsed Laser Deposition (PLD) with a multilayer protocol that introduces sequential Li_2_O enrichment during growth to produce ∼850 nm, polycrystalline LiMn_2_O_4_ cathodes for operation over a wider voltage window, in solid-state thin-film batteries. X-ray diffraction, Raman features, and Glow Discharge Optical Emission Spectroscopy depth profiles are consistent with the partial formation of a Li-rich spinel Li_2_Mn_2_O_4_ component during deposition. In our LiMn_2_O_4_/LiPON/Li cells, the wider voltage window cycling shows promising stability and provides capacities of 30–50 μAh·cm^–2^ at current densities of 1–30 μA·cm^–2^ corresponding to an approximately 2-fold increase in the capacity compared to the narrower voltage window.

## Methods

### Thin-Film Fabrication

Commercially available ceramic targets of LiMn_2_O_4_ and Li_2_O (CODEX) were employed for the Large-Area Pulsed Laser Deposition (LA PLD-5000, PVD Products) system, equipped with a Coherent (Lambda Physik) COMPex Pro 205 KrF excimer laser (λ = 248 nm). Both targets were sequentially ablated following a multilayer deposition approach, similar to our previous studies.^12,25–29^ For this work, the deposition parameters were adjusted to enhance lithiation and optimize processing time. Specifically, a 4:3 ratio of ablation pulses was maintained between the LiMn_2_O_4_ and Li_2_O targets, with 2600 and 2000 pulses, respectively, and a total of 60 repetition cycles. The deposition was conducted at a temperature of 650 °C and a partial oxygen pressure of 20 mTorr, with a target-substrate distance of 90 mm. The laser was operated at a fluence of 1.3 J·cm^–2^ and a frequency of 20 Hz to reduce total deposition time while minimizing excessive thermal exposure. The substrates used were 1 × 1 cm^2^ Pt (80 nm)/Ti (10 nm)/Si_3_N_4_ (300 nm)/SiO_2_ (100 nm)/Si (0.5 mm) chips, fabricated at the Institute of Microelectronics of Barcelona (IMB-CNM-CSIC). These multilayer depositions resulted in films with a total thickness of approximately 850 nm, as determined by Spectroscopic Ellipsometry and cross-sectional SEM imaging. This increased thickness and optimized deposition protocol were designed to achieve higher lithiation levels by incorporating additional lithium during the growth process.

Lithium–phosphorus oxynitride (LiPON) solid-electrolyte of 1 μm in thickness was manufactured by RF magnetron cosputtering of 2″ targets of Li_3_PO_4_ (99.95%, Kurt J Lesker Co., rate approximately 0.7 nm min^–1^) and Li_2_O (99.9%, Toshima Manufacturing, rate approximately 0.6 nm min^–1^) in a N_2_ atmosphere (flow set to 50 SCCM) at powers of 100 and 120 W, respectively, and a working pressure of 4 × 10^–3^ mbar. The target-to-substrate distance was set to 25 cm. The Li-metal anode for the solid-state configuration was thermally evaporated from lithium pellet (99+%, Thermo Fisher Scientific, Nexdep evaporator) at a rate of 25 Å s^–1^, to a total thickness of 2 μm. The Li deposition was patterned with circular dots of 0.0079 cm^2^ area as individual Li metal reservoirs that defined the separate cells.

### Structural Characterization

Scanning electron microscopy (SEM) was conducted at IREC using a Zeiss Auriga instrument equipped with a 30 kV Gemini FESEM column and an in-lens detector, enabling high-resolution imaging of the film’s cross-sectional and surface morphology. X-ray diffraction (XRD) measurements were performed using a Bruker D8 Advance diffractometer in the θ–2θ configuration, covering a range of 10–60° with a step size of 0.01°. To protect the detector, the region around the main reflection of the platinum substrate was excluded from the measurements. Glow Discharge Optical Emission Spectroscopy (GDOES) was performed using a Spectruma GDA 750 HR instrument equipped with a 2.5 mm anode. Measurements were conducted under optimized conditions of 700 V and 2.5 hPa Ar pressure to ensure a flat and uniform crater for accurate depth profiling. Raman spectroscopy was conducted using an Xplora Nano spectrometer (HORIBA) with 532 nm (green) and 633 nm (red) lasers. Spectra were acquired with an acquisition time of 2 s and 60 accumulations. Laser powers of 0.9 and 9 mW were used for the green laser, while 0.4 and 4 mW were used for the red laser, ensuring measurements below the threshold for sample degradation.

### Electrochemical Characterization

For liquid electrolyte measurements, the films were characterized using a TSC Surface Cell (rhd instruments, Germany). The electrolyte consisted of 1 M LiPF_6_ in a 50/50 (v/v) mixture of ethylene carbonate (EC) and dimethyl carbonate (DMC), both of battery-grade quality (Sigma-Aldrich). Lithium metal served as both the reference and counter electrode, while the active area of the film was defined by the cell (and empirically measured under optical microscope) as 0.3 cm^2^. The mass of the cathode was estimated based on thickness measurements obtained via Spectroscopic Ellipsometry. The film consisted of a dense 730 nm layer and a porous 117 nm layer (50% porosity), corresponding to an equivalent LiMn_2_O_4_ loading of 0.27 mg·cm^–2^, estimated using the theoretical density of LiMn_2_O_4_ (4.02 g·cm^–3^). For the solid-state configuration measurements, the liquid cell capacities were used as a starting point for C-rate determination. Therefore, 1C rate was defined as 30 μA cm^–2^ regardless of the voltage window. Cycling and EIS measurements for the solid-state setups were performed on Squidstat Plus potentiostats. Briefly, EIS was done in potentiostatic mode with 50 mV excitation amplitude, 10 steps per decade, at 3.5 V. Frequency range was set to 1 MHz–0.01 Hz.

## Results and Discussion

### Structural Characterization

Cross-sectional SEM images (Figure 1a) of PLD-deposited 850 nm-thick LiMn_2_O_4_ films on top of Pt-coated silicon substrate reveal a rough surface morphology, characteristic of the multilayer deposition process used for their fabrication.^25,26,28,29^ Using spectroscopic ellipsometry,^27,28,30^ the total thickness of the films was measured to be 847 nm, consisting of a dense 730 nm layer and a porous 117 nm top layer. For the solid-state thin-film batteries, LiPON was conformally deposited on the LiMn_2_O_4_ films (RF sputtering, see details elsewhere^31,32^ and in the Methods section). This conformal coverage (Figure 1a) indicates that the surface roughness remains within acceptable limits for device fabrication. The final solid-state batteries were completed with an evaporated layer of Li metal (∼2 μm) as the anode (not shown).

**Figure 1:**
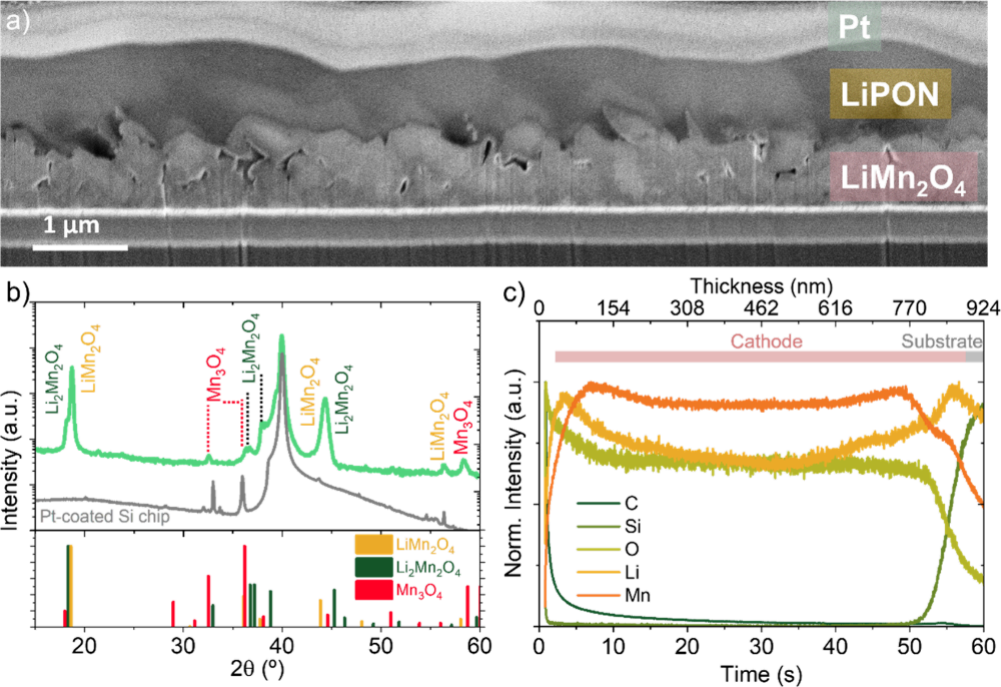
(a) Cross-sectional image of a LiMn_2_O_4_/LiPON/Pt film. Please note that the top Pt was deposited for ease of SEM visualization and LiPON protection. (b) XRD patterns of the as-deposited film and the bare Pt-coated chip as reference. The bottom panel shows references for LiMn_2_O_4_ (JCPDS 00-035-0782), Li_2_Mn_2_O_4_ (JCPDS 00-038-0299), and Mn3O4 (JCPSD 01-080-0382). (c) GDOES depth profile of the as-deposited film. GDOES profiles are semiquantitative; interface regions can be affected by sputtering/matrix effects.

XRD (Figure 1b) confirms polycrystalline LiMn_2_O_4_ as the dominant phase. In addition to the expected reflections, extra peaks appear at 2θ ≈ 18 and 37°, consistent with the (101) and (211) planes reported for tetragonal Li_2_Mn_2_O_4_. These features suggest the coexistence of the Li-rich spinel within the film. We note that this response coincides with adjustments to the PLD fabrication sequence (higher repetition rate and increased pulses per layer) implemented to shorten deposition time and increase Li supply during growth (see Methods). The modified sequence enables thicker films (∼850 nm) while preserving comparable surface roughness to our prior multilayer reports while shortening fabrication time.^25,26,28,29^ Minor Mn_3_O_4_ signatures are also observed (log-scale intensity), indicating limited surface reconstruction under the present conditions.

Glow Discharge Optical Emission Spectroscopy (GDOES) was used to probe the compositional depth profiles of the films (Figure 1c). Mn and O profiles remain relatively uniform throughout the film, while the Li signal shows a relative enrichment within the first ∼150 nm from the surface and the last ∼250 nm near the substrate. This means that ∼40 to 50% of the total thickness exhibits a higher Li concentration than the middle region of the film. Given the semiquantitative nature of the presented GDOES measurements, we interpret these trends as relative enrichment zones rather than absolute compositions. Taken together, the XRD and GDOES signatures support a nonuniform distribution of a Li-rich spinel component, with higher prevalence near the film boundaries. Additionally, carbon signal detected at the very surface (within the first 15–20 nm) likely corresponds to a thin surface carbonate layer formed during brief air exposure prior to the GDOES measurement.

Raman spectroscopy provides complementary evidence for a Li-rich spinel component and helps correlate compositional variations with depth. Spectra were collected with green (532 nm) and red (633 nm) lasers. The complementary use of the red laser enhances the detection of secondary Mn_3_O_4_ phases, a common feature usually occurring in LiMn_2_O_4_.^29^ The Raman spectra in Figure 2 are dominated by the LiMn_2_O_4_ bands (fittings and peak assignments are provided in the Supporting Information,^29,33^ and show a feature at ∼409 cm^–1^ that has been assigned to Li_2_Mn_2_O_4_ in literature.^33^ This band was not observed in earlier films grown by our standard sequence and is consistent with the increased lithiation associated with the modified PLD protocol. The Li_2_Mn_2_O_4_-related band is present at both low and higher laser powers (more prominent with 532 nm, see Supporting Information, Section II), suggesting that the Li-rich spinel extends to the near-surface region and is also present near the substrate, in agreement with the observed GDOES enrichment zones. Beyond the 409 cm^–1^ signature, we observe power- and wavelength-dependent changes in the Mn–O vibrational region (∼600 to 650 cm^–1^). Because 532 and 633 nm excitations probe slightly different effective depths and resonance conditions, the altered band ratios are consistent with a depth-varying convolution of LiMn_2_O_4_and Li-rich spinel modes, i.e., lithium distribution inhomogeneities across the film thickness.

**Figure 2:**
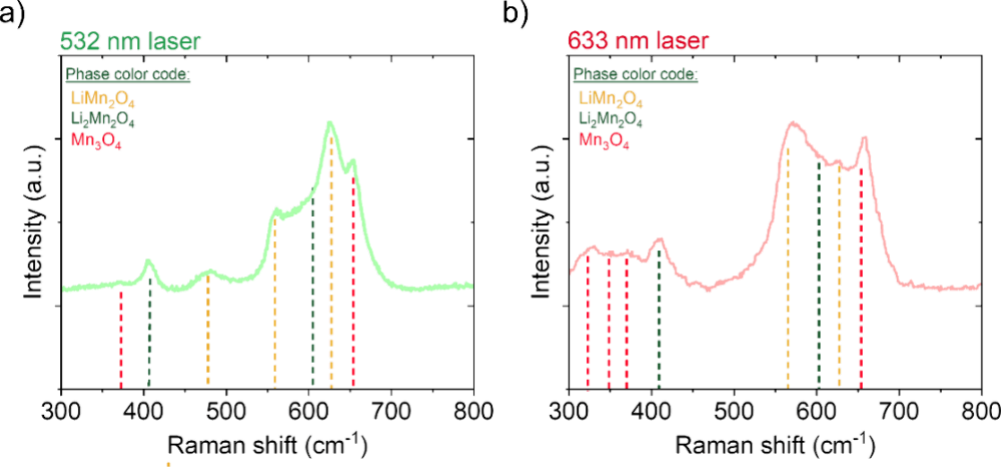
Raman spectra acquired with (a) green laser (532 nm) and (b) red laser (633 nm), acquired at 0.9 and 0.4 mW powers, respectively.

### Electrochemical Characterization

To evaluate the electrochemical performance of the PLD LiMn_2_O_4_ films across the wider voltage range, both liquid electrolyte (1 M LiPF_6_ in 50:50 v/v EC:DMC, Li metal) and solid-state (LiPON/Li metal) configurations were tested. Figure 3a shows the results of cycling in organic liquid electrolyte within the narrower voltage range (3.5–4.5 V), which revealed the characteristic redox plateaus of LiMn_2_O_4_. Notably, the initial open-circuit voltage (OCV) of the film was ∼3 V, closer to the expected OCV of Li_2_Mn_2_O_4_ rather than LiMn_2_O_4_ (∼3.5 V). Additionally, the first charge (shown in purple) displayed evidence of a lithium extraction process near 3 V, consistent with the presence of the Li_2_Mn_2_O_4_-like component. Importantly, RF-sputtered LiPON can partially lithiate the underlying oxide cathode, slightly increasing the Li extracted on the first charge in the solid-state cells relative to the liquid-electrolyte one. Unless stated otherwise, capacities are reported as true areal values; derived gravimetric estimations are based on the measured loading, given in the Methods.

**Figure 3:**
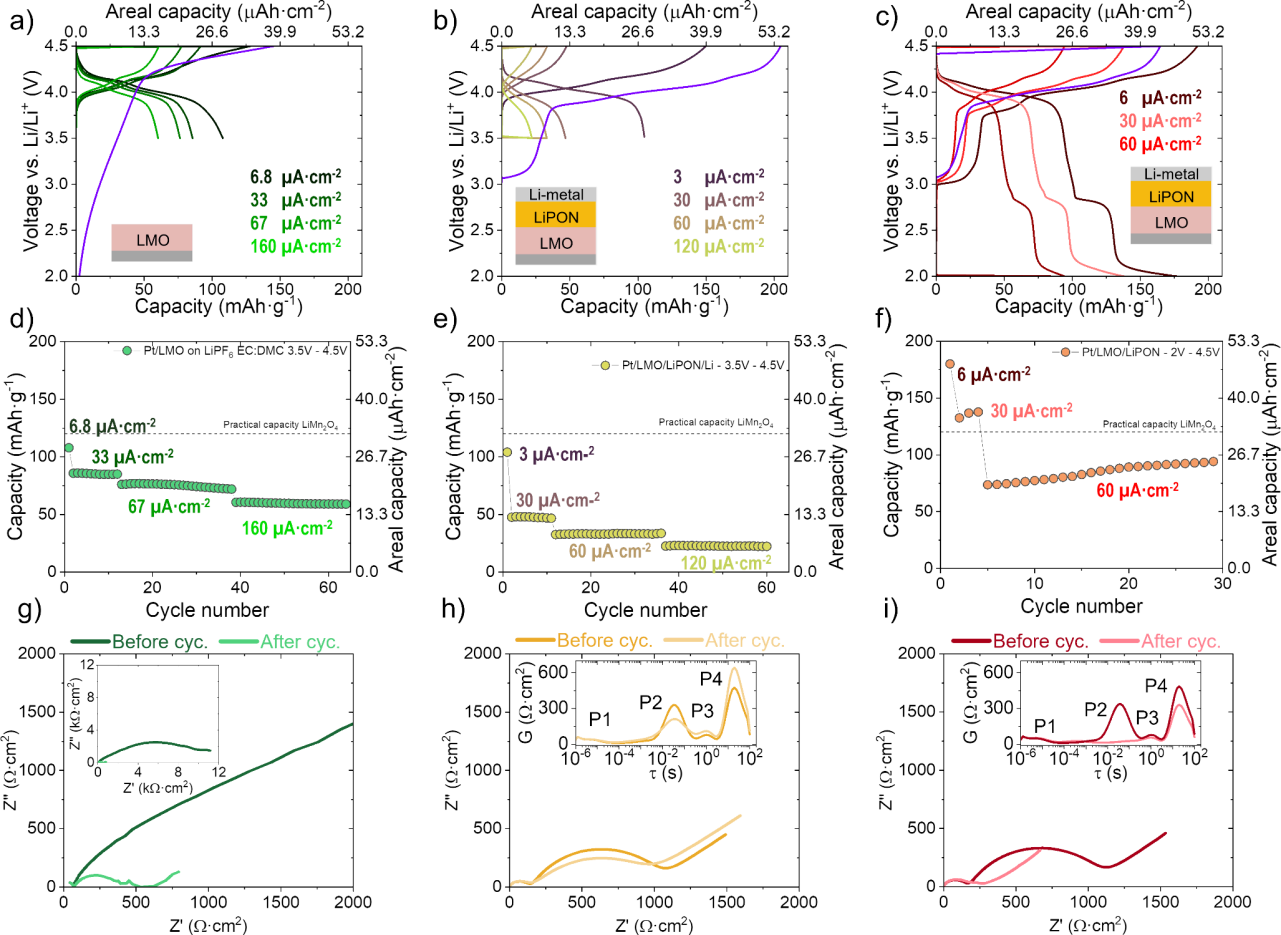
(a–c) Galvanostatic cycling of LiMn_2_O_4_ in liquid organic electrolyte (narrower window) and in solid-state cells of LiMn_2_O_4_/LiPON/Li (narrower and wider windows), respectively. (d–f) Extracted discharge capacities for the corresponding galvanostatic cycling. (g–i) EIS before and after cycling for each of the cells. Insets show the full EIS spectra for the liquid electrolyte cell and DRT analysis of the solid-state cells, respectively. Capacities are true areal values. EIS spectra were acquired at ∼4.0 V after the indicated protocols to enable comparison.

In organic electrolyte at low current densities within the narrower window (3.5–4.5 V), the films achieved gravimetric capacities close to the practical LiMn_2_O_4_ values reported, reaching device areal capacities of ∼27 and ∼14 μAh·cm^–2^ (107 and 59 mAh·g^–1^) at 6.8 and 160 μA·cm^–2^, respectively (Figure 3d). Cycling was stable under these conditions. However, extending cycling to the wider voltage window introduces the well-known Mn dissolution limitations, typically only mitigated in very thin films (<200 nm thick).^28^ Electrochemical impedance spectroscopy (EIS) shows an initial total impedance of ∼11 kΩ·cm^–2^, decreasing to ∼600 Ω·cm^–2^ after the cycling protocol between 3.5 and 4.5 V (Figure 3g). This drop is consistent with an interface improvement, such as removal of resistive surface species). This pronounced decrease is consistent with early interfacial conditioning/activation (e.g., improved wetting/contact and/or evolution of the cathode–electrolyte interphase), rather than a continuous monotonic performance increase over long times. One plausible contributor to this behavior is the dissolution of the surface carbonate layer, whose presence is suggested by the transient carbon signal observed in GDOES. Alternatively, recent work by Ou et al.^34^ suggests that LiMn_2_O_4_ cathodes in carbonate-based electrolytes form a dynamic cathode–electrolyte interphase, whose continuous restructuring can improve charge transfer. While this improvement suggests a reduction in film resistance during cycling, the issues at lower voltages exemplify the limitations of widening the useful cycling range of LiMn_2_O_4_ films in conventional liquid cells.

The electrochemical performance of the LiMn_2_O_4_ films in solid-state configuration was evaluated within both the narrower and wider voltage ranges as shown (Figure 3b,c). In both cases, the precycle (in purple) exhibits a ∼3 V plateau, with the initial OCV also close to ∼3 V. Although this behavior is typical of cathodes coated with a LiPON layer, which has been reported to lithiate the cathode underneath during deposition,^35^ in our case, the structural and electrochemical evidence from the liquid electrolyte cells support prelithiation during the PLD process itself. This prelithiation likely contributes to the Li-rich spinel footprints observed and to subsequent performance upon cycling.

As seen in Figure 3e, LiMn_2_O_4_ films show stable cycling in the narrower range (3.5 −4.5 V). At low current densities, areal capacities are comparable to those measured in liquid electrolyte of ∼27 μAh·cm^–2^. However, the C-rate capability is worse for the solid-state configuration, highlighting diffusion-limited behavior for our ∼850 nm planar films. In the liquid case, there is likely electrolyte infiltration into porosity within the film, which aids in the ionic transport, accounting for the differences between the configurations. This emphasizes the challenges associated with Li^+^ diffusion in thicker cathode films (as opposed to LiCoO_2_), where long diffusion paths can limit the cycling kinetics for LiMn_2_O_4_ without any compositional tuning that could increase its ionic diffusion in the bulk.

When the voltage range is widened to lower cutoff voltage of 2.5 V (Figure 3c,f), several effects appear. At very low current densities, the films achieved areal capacities of ∼30 to 50 μAh·cm^–2^, markedly higher than for the narrower voltage window. Interestingly, we observe an increase in capacity over the initial cycles, suggesting a progressive stabilization of the cathode–solid electrolyte interphase during early cycling or beneficial restructuring of the cathode layer. Similar behavior has been reported in micron-sized Al-doped LiMn_2_O_4_ samples cycled at low voltages, although those exhibited lower capacities overall.^9^ This interface improvement is corroborated by EIS: in the narrower window the total resistance shows no substantial change (Figure 3h), whereas in the wider window it decreases from ∼1200 to ∼250 Ω·cm^–2^ after cycling (Figure 3i), consistent with an improved transport within the electrode and at the interface.

To further examine these changes, Distribution of Relaxation Times (DRT) analysis was performed in the solid-state configurations (insets, Figure 3h–i). Four processes were tentatively identified^36,37^ : P1, likely associated with bulk LiPON resistance; P2, related to the cathode–electrolyte interface; P3, linked to the LiPON/Li metal interface; and P4, corresponding to charge transfer and ionic diffusion within the LiMn_2_O_4_ layer. In the narrower window, P2 showed a moderate decrease, while P4 increased, suggesting modest improvement at the CEI but some degradation in the cathode transport. In contrast, in the wider window P2 was strongly reduced and P4 also decreased, consistent with a reduced impedance contribution associated with the cathode–electrolyte interface and cathode transport/charge-transfer processes. While these assignments are consistent with prior studies,^36,37^ the physical origin of the relaxation processes arising from the DRT analysis in thin film solid-state batteries remains under discussion, as charge-transfer phenomena may involve both interfacial and bulk contributions in such compact geometries. Long-term cycling and detailed interfacial characterization, particularly through operando experiments, is the subject of ongoing work.

These findings highlight the importance of interfacial stability in solid-state battery operation. Xia et al. reported LiMn_2_O_4_/LiPON interfaces that evolve into an inactive Mn_3_O_4_/Li_2_O phase during cycling, accompanied by Mn diffusion into the LiPON.^24^ In contrast, our films exhibit a substantial impedance decrease and improved capacity in the wider window. Overlithiation during PLD, leading to a partial Li_2_Mn_2_O_4_-like component and the presence of cationic defects in our films may help mitigate such degradation. By accommodating volume changes and reducing interfacial stress, this phase could help preserve contact and transport, and suggests that cathode pre-engineering could be a suitable approach toward robust, high-performance solid-state LiMn_2_O_4_ systems.

As summarized in Figure 4a, at very low current densities (<10 μA/cm^2^) and within the narrower window, our films approach in both liquid and solid-state configurations equivalent gravimetric capacities close to the practical values of LiMn_2_O_4_ (∼120 mAh·g^–1^). The rate dependence differs, with solid-state capacities declining more rapidly due to transport limitations in ∼850 nm planar films. Liquid cells can partly offset this via electrolyte access to porosity. Extending cycling to include the ∼3 V plateau in the wider window compensates for these limitations, yielding significantly higher areal (and derived gravimetric) capacities. In this wider window, films reach >50 μAh·cm^–2^ at low current densities. Even at ∼(∼50 to 100 μA·cm^–2^, capacities approach the practical limits of the narrower-window counterparts. While some earlier reports show ≥80 μAh·cm^–2^, reproducibility has been challenging, likely reflecting stoichiometry control issues in RF sputtering.^23,38^

**Figure 4:**
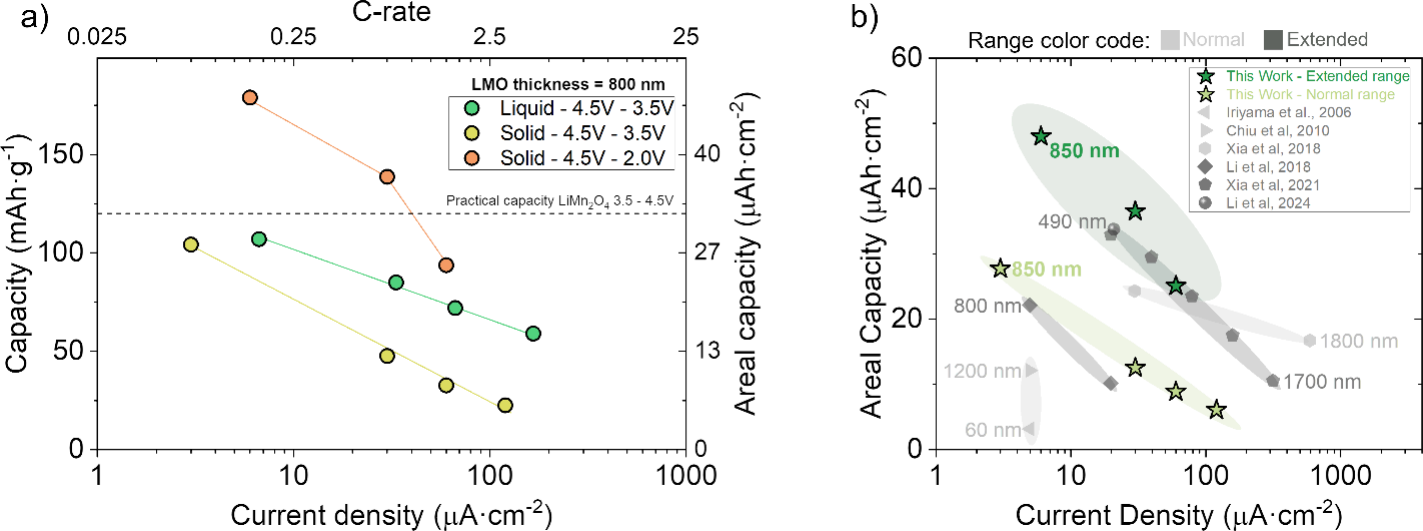
(a) Summary of capacities as a function of current density for the three configurations tested. (b) Comparison of the results of this work with the literature on Li–Mn–O systems integrated into solid-state thin-film batteries.

Our results highlight a key advantage of the solid-state configuration in the wider window: the absence of Mn dissolution that limits liquid cells. While the exact mechanisms remain under study, we suggest that the partial Li_2_Mn_2_O_4_ formation during fabrication may help accommodate volume changes during deep (de)­lithiation, contributing to mechanical stability and preserving interfacial contact. Additionally, cation vacancies, as proposed in our earlier works,^28,29^ could aid strain accommodation. Together, these features may enable a more robust and conductive interface for solid-state LiMn_2_O_4_-based thin film cathodes operating in the wider voltage window.

To contextualize these results, Figure 4b compares our data with representative reports over the past two decades of Li–Mn–O thin film device integration, based on the literature summarized in Table S1 in the Supporting Information. The plot shows that our films are competitive within device-class thickness even against thicker films or 3D architectures. These observations suggest that incorporating a Li-rich spinel component during fabrication can be a practical route to stabilize polycrystalline LiMn_2_O_4_ films for wider window operation. This approach can be further optimized to enable higher energy and power density thin-film LiMn_2_O_4_ for more sustainable microbatteries.

## Conclusions

This work demonstrates the successful integration of polycrystalline LiMn_2_O_4_ thin films with LiPON solid electrolytes using PLD. Through a multilayer deposition approach, partial incorporation of a Li-rich spinel (Li_2_Mn_2_O_4_-like) component was achieved during film growth. Structural, electrochemical data and comparison toward literature suggest that this phase might contribute to stable cycling in the wider voltage window. As a result, areal capacities of ∼30 to 50 μAh·cm^–2^ were obtained.

Wider-window cycling also coincided with a progressive decrease in interfacial and bulk resistances (EIS/DRT), in contrast to liquid organic electrolytes wherein Mn dissolution limits extension to lower voltages. The interface appears to improve during early cycling in the wider window, potentially benefiting from the Li-rich spinel and the films’ cation-vacancy landscape, which may help accommodate volume change. Reduced impedance accompanies a gradual capacity increase over initial cycles.

Despite these advances, diffusion-limited kinetics remain a challenge for thick-film architectures, especially at higher current densities. Future work should aim to unravel the mechanisms underlying interface rearrangement during long-term cycling and explore ways to further optimize ion transport within device-class thickness.

## Supplementary Material



## Data Availability

Data for this article will be available at IREC’s institutional repository, hosted in Zenodo at https://zenodo.org/communities/irec/.

## Notes

Literature survey of representative solid-state Li–Mn–O thin-film battery reports (Table S1); Raman spectroscopy details, including power-dependent and wavelength-dependent spectra, peak fittings, and phase identification for LiMn_2_O_4_, Li_2_Mn_2_O_4_, and Mn_3_O_4_ (Figures S1–S3) (PDF)
