# Supplementary material for: Pulsed-Laser-Deposited LiMn2O4 Thin-Film Solid-State Microbatteries with Extended Voltage Window Cycling
Source: ACS Appl Energy Mater. 2026 Mar 17;9(7):3893–900. doi: 10.1021/acsaem.5c03983 (PMC13080773; doi:10.1021/acsaem.5c03983)
Supplement: Supplementary file 1 [file ae5c03983_si_001.pdf]

Supporting Information

Pulsed-laser-deposited $LiMn_2O_4$ thin film solid-state microbatteries with extended voltage window cycling

Juan Carlos Gonzalez-Rosillo*†1, Jędrzej Morzy*2, Yaroslav E. Romanyuk2, Moritz H. Futscher2, Albert Tarancón†1,3, Alex Morata†1.

1. *Department of Advanced Materials for Energy Applications, Catalonia Institute for Energy Research (IREC), Jardins de les Dones de Negre 1, 08930, Sant Adrià del Besòs, Barcelona, Spain*
2. *Laboratory for Thin Films and Photovoltaics, Empa – Swiss Federal Laboratories for Materials Science and Technology, Überlandstrasse 129, 8600 Dübendorf, Switzerland.*
3. *Catalan Institution for Research and Advanced Studies (ICREA), Passeig Lluís Companys 23, 08010, Barcelona, Spain*

**: shared first co-authorship*
†: corresponding authors: jcgonzalez@irec.cat; atarancon@irec.cat; amorata@irec.cat

Section I: Literature survey of solid-state $LiMn_2O_4$ thin film batteries

Table S1 compiles representative studies on the integration of $LiMn_2O_4$ in solid-state thin-film batteries over the past 25+ years. The table includes fabrication methods, processing conditions, film thicknesses, voltage ranges, cycling performance, and reported areal capacities. It is organized chronologically to reflect the evolution of materials design and integration strategies, from early evaporated and sputtered films to more recent developments in nanostructured and composite architectures. Because areal capacity scales with thickness (ideally) and protocols (window, rate, porosity, active area) vary across reports, cross-study comparisons should be interpreted cautiously. This overview serves as a reference point for benchmarking our results and highlights the continued challenge of achieving high areal capacities (particularly for planar films with thickness ≥ 1 μm) while maintaining stable cycling and reproducibility.

Device structure	Fabrication Method	Deposition Temperature	Crystalline/ Amorphous	Cycling Range	Number of Cycles	Film Thickness	Areal Capacity (μAh·cm^{-2})	Year and Ref.
Li/LiPON/ $LiMn_2O_4$	1) e-beam evaporation and 2) RF magnetron sputtering	1) RT + post-annealing at 800 ºC and 2) RT	1) Crystalline and 2) amorphous	1) 3.8 - 4.5V and 2.5 - 4.5V and 2) 2.5-4.5V	not specified	1) 300 nm and 2) 660 nm LMO, 1 μm LiPON	1) 9.6 μAh·cm^{-2} at 10.8 μA·cm^{-2} (range 3.8 - 4.5V) and 14 μAh·cm^{-2} at 16.5 μA·cm^{-2} and 2) 32 μA·cm^{-2} at 2 μA·cm^{-2} and 14 μA·cm^{-2} at 40 μA·cm^{-2}	1995,ref. [1]
Li/LiPON/ $LiMn_2O_4$	RF Magnetron sputtering	< 100 ºC	Nanocryst.	2.5 - 4.5 V	3400	1) 270 nm and 2) 2500 nm LMO, 1 μm LiPON	1) 11 μAh·cm^{-2} at 40 μA·cm^{-2} and 2) 81 μAh·cm^{-2} at 2 μA·cm^{-2}	1999,ref. [27]
Li/LiPON/ $LiMn_2O_4$	RF Magnetron sputtering	RT + 750 ºC postannealing in RTA	Crystalline	3.7 - 4.3 V	not specified	300 nm LMO, 1 μm LiPON	15, 14.4, 12.6 μAh·cm^{-2} at 50, 100 and 800 μA·cm^{-2} respectively	1999,ref.[2]
V_2O_5/ LiPON/ $LiMn_2O_4$	RF Magnetron sputtering	RT	Amorphous	0.3 – 3.5 V vs. V_2O_5	40	800 nm LMO, 1 μm LiPON, 200-250 nm V_2O_5	Best reported - 18 μAh·cm^{-2} at 2 μA·cm^{-2}	2001,ref. [3]
Li/LiPON/ $LiMn_2O_4$	RF magnetron sputtering	RT + 550 ºC postannealing	Crystalline	3.0-4.5V	not specified	2000 nm LMO, 1 μm LiPON	102, 84, 62, 35 and 12 μAh·cm^{-2} at 10, 500, 1000, 1500 and 2000 μA·cm^{-2}	2005,ref. [4]
Li/LiPON/ $LiMn_2O_4$	Pulsed Laser Deposition	700 ºC	Crystalline	3.5 - 4.25 V	500	60 nm LMO	3 μAh·cm^{-2} at 5 μA·cm^{-2}	2006,ref. [5]
SnO2/ LiPON/ $LiMn_2O_4$	RF magnetron sputtering	RT + 600 ºC postannealings + anneal the	Crystalline	1.5 - 3.9V	50	1200 nm LMO, 1 μm LiPON	12 μAh·cm^{-2} at 5 μA·cm^{-2}	2010,ref. [6]

		LMO/LiPON at 200 ºC						
3D $LiMn_2O_4$ nanowall/ LiPON/Li	DC Magnetron Sputtering	300 ºC/600 ºC + postannealing at 700 ºC	Crystalline	3.3-4.4V	400 cycles	1.8 µm (3D LMO nanowalls), 2 µm LiPON	24.2 and 16.6 $\mu Ah\cdot cm^{-2}$ at 30 and 600 $\mu A\cdot cm^{-2}$	2018,ref. [7]
ZnO/ LiPON/ $LiMn_2O_4$	RF Magnetron sputtering	Room temperature	Amorphous	0.5-5.0V	50 cycles	LMO: 800 nm, LiPON: 500 nm, ZnO: 450 nm	22 and 10 $\mu Ah\cdot cm^{-2}$ at 5 and 20 $\mu A\cdot cm^{-2}$	2018,ref. [8]
3D Li_xMnO_2/ LiPON/Li	Reactive DC sputtering	RT + 180 ºC annealing after LiPON dep.	Mixed	2.0-4.3V	1000 cycles	1.7 µm	32.8, 29.4, 23.4, 17.4, and 10.4 $\mu Ah\cdot cm^{-2}$ at 20, 40, 80, 160, and 320 $\mu A\cdot cm^{-2}$	2021, ref. [9]
Li_2MnO_3/ LiPON/Li	RF magnetron sputtering	300 ºC + 800 ºC post-annealing	Crystalline	2.0-4.8V	1000 cycles	490 nm	33.7 $\mu Ah\cdot cm^{-2}$ at 21 $\mu A\cdot cm^{-2}$	2024, ref. [10]
**Mixed $LiMn_2O_4$-$Li_2Mn_2O_4$/ LiPON/Li**	**LMO: Pulsed Laser Deposition LiPON: RF sputtering**	**650ºC**	**Crystalline**	**2.0 - 4.5 V**	**30**	**850**	**48, 36, 25 $\mu Ah\cdot cm^{-2}$ at 6,30,60 $\mu A\cdot cm^{-2}$**	**2025, This Work**

Table S1: Literature survey of solid-state $LiMn_2O_4$-based thin film batteries

Section II: Raman Spectroscopy

Raman spectroscopy was employed to provide evidence consistent with the presence of the $Li_2Mn_2O_4$ phase and assess phase distribution across the film depth. Spectra were acquired using both 532 nm (green) and 633 nm (red) lasers at two laser power levels, carefully selected to avoid degradation of the films, and to introduce depth sensitivity[11] due to the relatively thick nature of the film (~850 nm), Fig. S1. The Raman spectra reveal distinct vibrational modes that are commonly assigned to specific phases based on well-established literature.[12,13] The dominant peaks correspond to the $LiMn_2O_4$ spinel structure. A key feature is the emergence of a peak at ~409 cm^{-1}, commonly reported as the chemical signature of the $Li_2Mn_2O_4$ phase, which had not been previously observed in our films fabricated using standard deposition conditions, Fig.S2 and S3.[12] This peak is detected at both low and high laser power levels and with both excitation wavelengths, with variations in relative band intensities. This is consistent with a Li-rich spinel contribution extending inhomogeneously through the film thickness. The use of the red laser enhances the detection of Mn_3O_4, a secondary phase commonly observed in $LiMn_2O_4$ films, particularly at the surface, and previously confirmed in our work using Tip-Enhanced Raman Spectroscopy.[13] At low laser power, the relative intensities of both Mn_3O_4 and $Li_2Mn_2O_4$ bands are enhanced, indicating a degree of inhomogeneity in phase distribution near the surface. These observations are in good agreement with the lithium signal enrichment seen in GDOES depth profiles (noting that Li quantification in this case is semi-quantitative). Peak fittings were performed using LabSpec (Horiba) after baseline subtraction and normalization to the most intense band. All spectra were fitted using Lorentzian functions, and the resulting deconvolutions and peak assignments are presented in Figures S2 and S3.

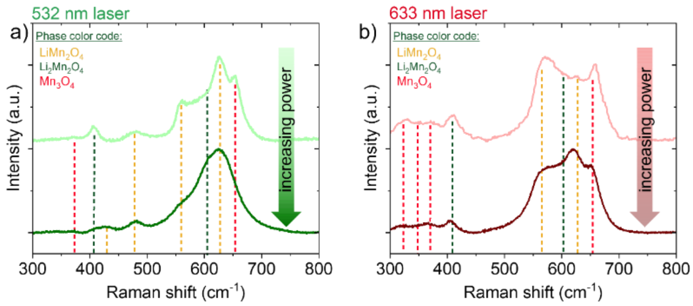

Figure S1: Raman spectra with acquired with a) green and b) red laser, with laser powers of 0.9 and 9 mW were used for the green laser, while 0.4 and 4 mW were used for the red laser, ensuring measurements below the threshold for sample degradation.

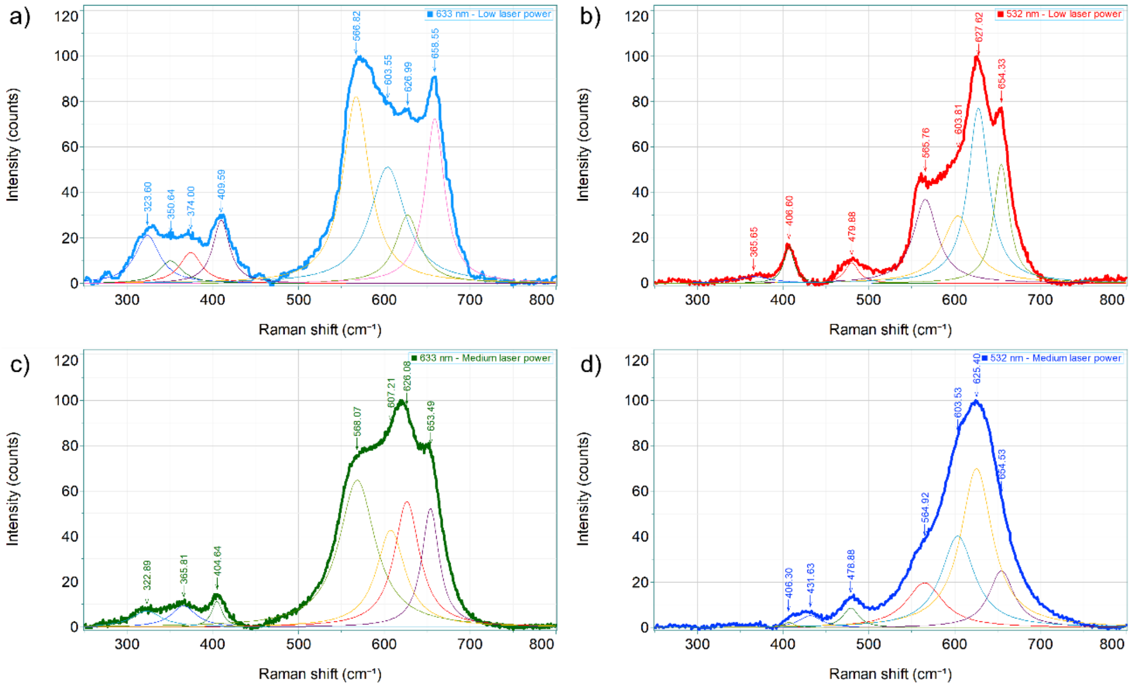

Figure S2: Fittings for the observed Raman spectra.

a) **633 nm - Low laser power**

Peak	p	a	w	ar	Phase ID
1	323.6	21.15	34.79	1056.61	(red)
2	350.64	9.93	31.7	464.37	(red)
3	374	13.7	30.6	625.15	(red)
4	409.59	27.85	23.4	989.78	(green)
5	566.82	82.37	37.41	4625.55	(yellow)
6	603.55	51.21	50.25	3787.09	(green)
7	626.99	30.04	34.06	1533.8	(yellow)
8	658.55	72.56	27.07	2959.01	(red)

b) **532 nm - Low laser power**

Peak	p	a	w	ar	Phase ID
1	365.65	2.98	47.33	203.73	(red)
2	406.6	16.48	14.13	358.42	(green)
3	479.88	9.09	19.5	271.86	(yellow)
4	565.76	36.94	34.04	1895.89	(yellow)
5	603.81	29.65	43.65	1921.43	(green)
6	627.62	77.1	29.42	3422.44	(yellow)
7	654.33	52.34	21.77	1732.17	(red)

c) **633 nm - Medium laser power**

Peak	p	a	w	ar	Phase ID
1	322.89	7.12	41.99	420.9	(red)
2	365.81	9.46	34.9	487.2	(red)
3	404.64	11.38	15.16	264.95	(green)
4	568.07	64.8	51.01	4878.93	(yellow)
5	607.21	42.78	40.56	2585.36	(green)
6	626.08	55.37	35.74	2960.43	(yellow)
7	653.49	52.26	24.58	1944.56	(red)

d) **532 nm - Medium laser power**

Peak	p	a	w	ar	Phase ID
1	406.3	2.34	15.23	54.85	(green)
2	431.63	5.33	32.18	258.05	(yellow)
3	478.88	8.69	22.89	303.93	(yellow)
4	564.92	19.7	52.25	1517.33	(yellow)
5	603.53	40.34	46.63	2781.5	(green)
6	625.4	70	44.53	4606.56	(yellow)
7	654.53	25.08	35.56	1327.27	(red)

Color code- Peak assignment

$LiMn_2O_4$ (yellow)	$Li_2Mn_2O_4$ (green)	Mn_3O_4 (red)

Figure S3: Peak fittings and peak assigment for each spectra

References

1 J. B. Bates, N. J. Dudney, D. C. Lubben, G. R. Gruzalski, B. S. Kwak, X. Yu and R. A. Zuhr, *J Power Sources*, 1995, **54**, 58–62.

2 Y. S. Park, S. H. Lee, B. Il Lee and S. K. Joo, *Electrochemical and Solid-State Letters*, 1999, **2**, 58–59.

3 M. Baba, N. Kumagai, N. Fujita, K. Ohta, K. Nishidate, S. Komaba, H. Groult, D. Devilliers and B. Kaplan, *J Power Sources*, 2001, **97–98**, 798–800.

4 N. J. Dudney, in *Materials Science and Engineering: B*, Elsevier BV, 2005, vol. 116, pp. 245–249.

5 Y. Iriyama, K. Nishimoto, C. Yada, T. Abe, Z. Ogumi and K. Kikuchi, *J Electrochem Soc*, 2006, **153**, A821.

6 K. F. Chiu, C. C. Chen, K. M. Lin, H. C. Lin, C. C. Lo, W. H. Ho and C. S. Jiang, *Vacuum*, 2010, **84**, 1296–1301.

7 Q. Xia, S. Sun, J. Xu, F. Zan, J. Yue, Q. Zhang, L. Gu and H. Xia, *Small*, 2018, **14**, 52, 1804149. DOI:10.1002/smll.201804149.

8 L. Li, S. Liu, H. Zhou, Q. Lei and K. Qian, *Mater Lett*, 2018, **216**, 135–138.

9 Q. Xia, Q. Zhang, S. Sun, F. Hussain, C. Zhang, X. Zhu, F. Meng, K. Liu, H. Geng, J. Xu, F. Zan, P. Wang, L. Gu and H. Xia, *Adv. Mater.*, **2021**, 33, 5, 2003524 DOI:10.1002/adma.202003524.

10 Q. Li, W. Liu, J. Wang, Q. Xia and H. Xia, *J Power Sources*, 2024, **602**, 234371, DOI:10.1016/j.jpowsour.2024.234371.

11 J. Kreisel, M. C. Weber, N. Dix, F. Sánchez, P. A. Thomas and J. Fontcuberta, *Adv Funct Mater*, 2012, **22**, 5044–5049.

12 C. M. Julien and M. Massot, *Materials Science and Engineering: B*, 2003, **100**, 69–78.

13 J. C. Gonzalez-Rosillo, M. Guc, M. O. Liedke, M. Butterling, A. G. Attallah, E. Hirschmann, A. Wagner, V. Izquierdo-Roca, F. Baiutti, A. Morata and A. Tarancón, *Chem. Mater*, 2024, **36**,12, 6144-6153 DOI:10.1021/acs.chemmater.4c00888.
